# Terpene-loaded Liposomes and Isopropyl Myristate as Chemical Permeation Enhancers Toward Liposomal Gene Delivery in Lung Cancer cells; A Comparative Study

**Published:** 2016

**Authors:** Mostafa Saffari, Farshad Hoseini Shirazi, Hamid Reza Moghimi

**Affiliations:** a *School of Medicine, Kashan University of Medical Sciences, Kashan, Iran. *; b *School of Pharmacy, Shahid Beheshti University of Medical Sciences, Tehran, Iran.*; c *Pharmaceutical Sciences Research Centre, Shahid Beheshti University of Medical Sciences, Tehran, Iran.*; d *Protein Technology Research CenterˏShahid Beheshti University Of Medicals Ciencesˏ Tehranˏ Iran.*

**Keywords:** Liposomal gene delivery, Penetration enhancer, Isopropyl myristate, Cineole, Limonene

## Abstract

Gene therapy is in its development stage as a novel method for cancer treatment. Liposomes look promising as gene delivery vectors; however, investigations have shown that these vesicles are not doing well in some cases. It was decided here to investigate the possibility of augmentation of liposomal gene delivery by chemical penetration enhancers.

Cationic liposome containing antisense oligonucleotide (AsODN) against lung cancer was prepared by ethanol injection method. Liposomal cineole and limonene (as enhancers) were prepared by film hydration method. Isopropyl myristate (IPM) was also investigated as penetration enhancer. Liposomes were evaluated for their size, zeta potential and encapsulation efficiency. Cancer cells (A549) were pretreated with liposomal terpenes prior to treatment with liposomal antisense or scrambled oligonucleotide. Cell viability was evaluated by MTT assay.

Oligonucleotide -containing liposome showed particle size of about115 nm and zeta potential of 0.6 mV. Liposomal cineole significantly (P<0.05) increased specific activity of liposomal antisense but limonene didn’t show such an effect. IPM increased both specific and non-specific cytotoxicity of Oligonucleotide.

These results show that penetration enhancers (such as cineole) may be used for improving liposomal gene delivery and to reduce non-specific toxicity. Concentration and chemical nature of enhancer has prominent effect in their efficacy.

## Introduction

Different investigations for development of more efficient strategies are in progress to target specific sub-cellular pathways associated with tumor growth (1). Among these, gene delivery has been of the most promising advances in novel generation of drug delivery (2). Gene delivery relies on the possibility of the genetic material, including oligonucleotides, to interact directly with specific intracellular targets (3). However, the delivery portion and stability of these agents is yet to be optimized for a successful therapy (4, 5). Nuclease degradation before action and the inability to penetrate cellular membranes are the major drawbacks of antisense oligodeoxynucleotides (ODN) (6). Therefore, suitable carriers and strategies are crucial in successful gene therapy.

In the present work, DOTAP cationic liposomes containing AP1261 as antisense oligonucleotide (AsODN) against protein kinase C-alpha (PKC-α) were prepared. PKC-α, is a target molecule and play an important role in cell regulation and proliferation (7), especially in A549 cells, as a non-small cell lung carcinoma (NSCLC) (8). Liposomes are one of the most common carriers used in drug delivery (9). However, liposomes still have some problems in cell uptake. It was decided here to investigate the effect of penetration enhancer on liposomal delivery (10). Previously we reported successful ODN delivery using urea as penetration enhancer. Here, we decided to use terpenes as chemical penetration enhancers in liposomal gene delivery. 

Terpenes are mostly lipophilic compounds and are impossible to be delivered by simple aqueous solutions. To exploit terpenes as chemical penetration enhancers, it is necessary to apply organic solvents, such as DMSO, to solve them in culture media. Organic solvents are often toxic and may interfere with their action, therefore it is better to be avoided due to their cytocidal effects and interference in experimental results (11). An alternative to organic solvents is liposome. Advantages of liposomal vehicles for essential oils has been shown before (12). It was decided here to formulate terpenes as nanoliposomes. 

Based on different reports in this issue, structure of enhancer has determining effect on magnitude of enhancement effect (13), therefore two terpenes with different chemical structures (limonene, a hydrocarbon and cineole, an ether) were chosen for this investigation. Among different methods, thin film hydration, that is the most widely used method for preparation of liposomes (14, 15), was employed here for preparation of liposomal terpenes. Finally, isopropyl myristate (IPM) that have permeation enhancement effect in transdermal drug delivery (16) was used here for comparison. IPM was dissolved in DMSO and used on cells.

## Experimental

*material*

Distearoyl phosphocholine (DSPC) were purchased from Northern Lipids (Vancouver, Canada). Egg phosphocholine were purchased from Lipoid (Germany). 1,2-dioleoyl-3-trimethylammonium-propane (DOTAP), cholesterol (Chol), bromophenol blue, polycarbonate filters, Sepharose DEAE (diethylaminoethanol), Triton X-100, 1,8-cineole, limonene, isopropyl myristate and HEPES were obtained from Sigma-Aldrich Chemical Company (St. Louis, USA). A 20-mer phosphorothioate modified AsODN 5’-TsCsCs AsTsGs AsCsGs AsAsGs TsAsCs AsGsCs CsGs-3’ directed against protein kinase C-α mRNA, and its disarranged sequences (ScODN, as control) were synthesized by Bioneer (Korea) and used as a previously-validated model of antisense therapy in non-small cell lung cancer (8). Fetal bovine serum was form Gibco BRL. All other reagents were of analytical grade. 

*methods*

*Preparation and characterization of liposomes*

*Preparation of ODN-containing liposomes by ethanol injection method*

Ethanol injection method was used as described previously (17). Briefly, lipid mixture, DOTAP/DSPC/Cholesterol/PEG2000-DSPE, was dissolved in absolute ethanol and this cocktail was injected gently under vigorous shaking into ODN solution in citrate buffer (pH=4). Liposomes were then dialysed against HEPES buffered saline (HBS, pH = 7.5) to remove ethanol from medium and adjust the pH. Residual free ODN were subsequently removed through Sepharose DEAE gel filtration chromatography. 

*Preparation of liposomal terpenes by thin layer film hydration*

Terpene**-**containing liposomes were prepared by thin film hydration method, with modification of Sinico method (12). Briefly egg phosphatidile choline (egg PC) /DOTAP/Chol /cineole or limonene (14:1:7:78 molar %) were dissolved in 10 mL chloroform and the solvent was evaporated in a rotary balloon and shacked for 2 h at 40 °C. The obtained lipid film was then hydrated with HBS (pH 7.4) to form initial liposomes at 40 °C. Liposomes were then extruded (3 times through 200 nm and 3 times through 100 nm) polycarbonate filter membranes (Millipore, USA) and subsequently purified by G50 Sephadex column to separate free terpenes.

*Particle size and zeta-potential determination*

Zeta potential and particle size of liposomes were determined by Malvern Zetasizer (UK). Liposomes were diluted with HBS. Measurements were carried out at 25 °C, under conditions of: viscosity, 0.88 cP; reflex index, 1.33. 

*ODN encapsulation efficiency*

ODN encapsulation efficiency was expressed as recovered ODN/lipid weight ratio compare to initial ODN/lipid ratio in each step. The recovery of ODN was determined separately by UV-spectrophotometer (Shimadzu, Japan) at 260 nm after liposome solubilization in chloroform/methanol (1:2.1) and based on a previous calibration curve. Phospholipid content was assayed via complex formation between phospholipids and ammonium ferrothiocyanate (17).

*Preparation of IPM solution*

Isopropyl myristate was dissolved in 1% DMSO in RPMI 1640 culture medium. The concentration of IPM in culture medium was set to be 5, 10, 20, 30 and 50 mg/mL.

*Cell culture studies*

A549 cells, obtained from Pasteur Institute (Tehran, Iran) were grown in RPMI 1640 medium supplemented with 10% heat-inactivated FBS, 100U/mL penicillin and 100 μg/mL streptomycin. Cells were maintained at 37 ^◦^C in a 5% CO_2_-incubator. Cell viability were evaluated either by either cell counting using 0.4% trypan blue solution under light-inverted microscope (Leica, Germany) or cytotoxicity studies by MTT assay.

*MTT assay*

10000 A549 cells were seeded in each well of 96-well plate and were incubated at 37 ^◦^C in a 5% CO_2_ for 24 h. The cells were then treated with different liposomal ODN concentration (either containing AsODN or ScODN), for 48 h. After that, 20 µL of MTT (5 mg/mL) were added to each well and incubated 4 h in incubator. The medium containing MTT was then aspirated and 100 µL DMSO was added to each well, and the plates were agitated gently until the MTT formazan had dissolved. The absorbance of each well was measured using a plate reader (Rainbow, Australia) at 570 nm, subtracting the absorbance at 650 nm as the reference (there is no absorbance by MTT at 570 nm). Cell viability was then calculated using measured absorbences.

*In-vitro evaluation of IPM and liposomal terpenes for their delivery-enhancement activity*

Best liposomal ODN concentration that showed most sequence specific effect, were chosen for enhancement studies. For enhancement studies and based on our prior experience (19), the cells were first treated for 4 h with IPM, liposomal cineole or liposomal limonene at 1-10µg/mL (in non-toxic ranges). The cell media containing enhancers were then removed and cells were treated with liposomal preparations containing either AsODN or ScODN at 150nM ODN in the culture medium for 48 h. After this period, sequence specific-antisense activity of liposomal ODNs was evaluated by MTT. The same experiments were performed in the absence of terpenes or IPM as control.

*Statistical analysis*

Comparisons of viabilities and liposomal properties among different groups were performed using one-way analysis of variance (ANOVA) accompanied with Tukey posthoc. Standard curves were generated based on linear regression and statistical significance was determined so values of P<0.05 was considered as significant.

**Table 1:** Characteristics of different prepared liposomes (data are mean ± SD, n = 3).

**Encapsulation** **efficiency** **(%)**	**Zeta potential** **(mV)**	**Polydispersity index**	**Size** **(nm)**	**Formulation**
87.5 ± 3.8	0.6 ± 0.7	0.14	115	Control (F1)
71 ± 4.08	6.1 ± 7.1	0.11	102	Limonene (F2)
67.7± 3.2	6.8 ± 0.6	0.15	128	Cineole (F3)

**Figure 1:**
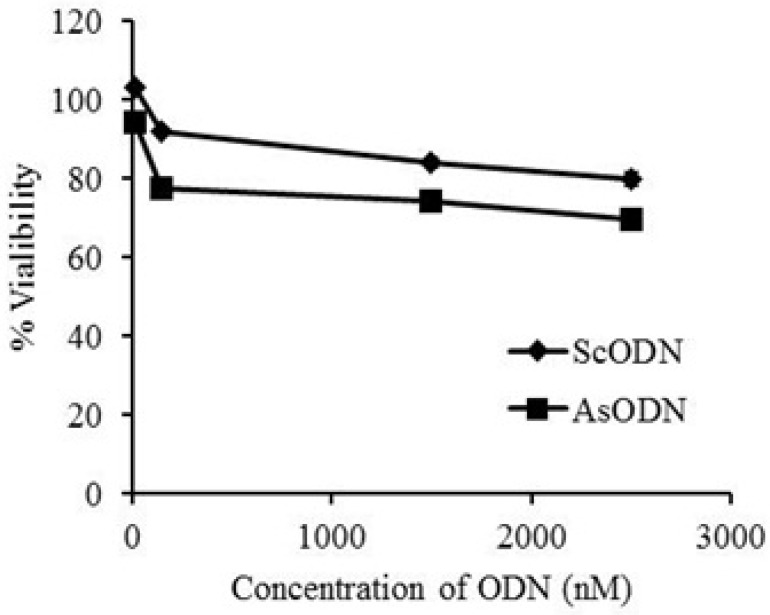
Antiproliferative effect of liposomal antisense oligonucleotide (AsODN) in comparison to its scrambeled control (ScODN) at different concentrations after 48 hours exposure time evaluated by MTT assay. Data are mean (n = 6

**Figure 2:**
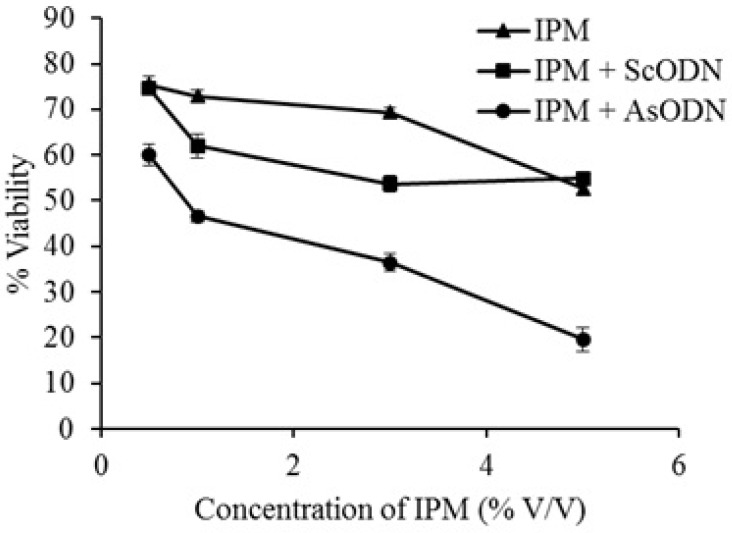
Effect of 4 hours isopropyl myristate (IPM) pretreatment on antiproliferative action of liposomal antisense oligonucleotide (AsODN) in comparison to its control (ScODN) at different concentrations. Data are mean ± standard error (n = 3

**Figure 3:**
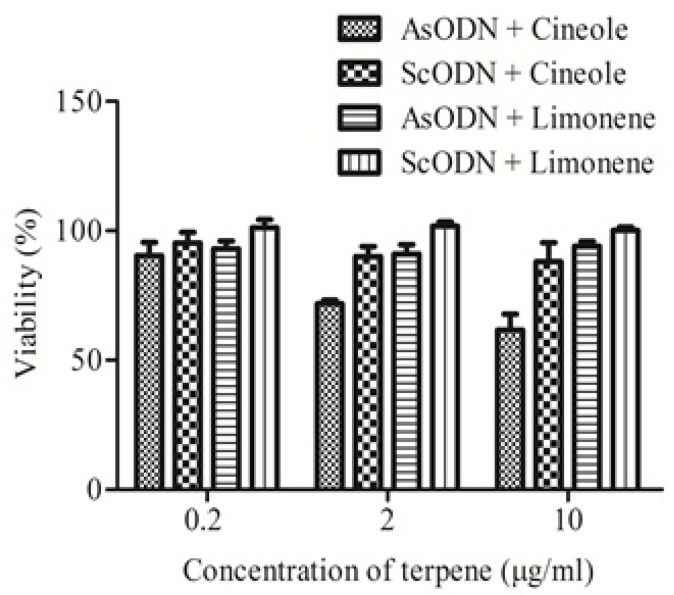
Enhancement effects of limonene and cineole on antiproliferative action of liposomal antisense oligonucleotide (AsODN) in comparison to its control (ScODN) at different concentrations. Data are mean ± standard error (n = 3

## Result and disscussion

*Characterization of liposomal formulations*

Table 1 shows size, zeta potential and encapsulation efficiency (EE) of prepared liposomes. The control liposomal formula was uniform and with particle size around 115 nm and approximately neutral at physiologic pH with the zeta-potential of 0.6 mV that didn’t differ from zero significantly (P>0.05). Encapsulation efficiency of this formulation was around 90% with suitable poly dispersity index (0.14) that was obtained without extrusion. 

Cholesterol, DSPC and DOTAP were incorporated in the liposome to entrap ODN actively (17). PEG2000-DSPE not only renders the liposome sterically stabilized for in-vivo application but also allows the control of liposome size.

For cineole-containing liposomes, size and zeta potential were 128 nm and 6.8 mV respectively. In agreement with Sinico *et al.* (12) EE of this liposome was around 70%. Limonene containing liposomes, showed particle size of 102 nm, positive charge of 6.1 ± 7.1 mV (18) and EE of more than 70% in agreement with cineole-containing liposomes and Sinico's work. The liposomes were stable during experimental period based on zeta potential and particle size measurement.

The reduced encapsulation efficiency in terpene-containing liposomes, in comparison to terpeneless liposomes, should be due to the involvement of lipophilic terpenes in the lipid bilayers. It has been shown that in spite of the hydrophilicity of ODNs, entrapment in the lipid bilayers plays an important role in their encapsulation (19). The same mechanism (reduced negatively-charged ODNs in the system) might be responsible for increased zeta potential of terpene-containing liposomes.

*Inhibition of cell proliferation by liposomal ODN*

The anti-proliferative ability of liposomal AsODN at a concentration range of 15 to 2500 nM against A549 cells was tested and compared with ScODN-containing liposomes. The inhibitory effect of ODNs on cell proliferation was determined after 48 h of transfection using the MTT assay. As shown in Figure 1. ScODNs had almost no significant effect on the proliferation of A549 cells. But, liposomal AsODNs reduced cell viability significantly and brought about an inhibition activity of 20% in A549 cells. At 150 nM concentration of ODN, difference between AsODN and ScODN (which is defined as sequence specific inhibitory effect) was at its maximum and after this point; it seems that non-specific toxicity of ScODN is increased. This is in agreement with Tamaddon (19) who showed that non-specific cytotoxicity increases by increasing the concentration. Therefore, the 150 nM of ODN concentration was chosen for enhancement studies.

The present results demonstrated that liposomal carriers are able to promote the transfection of AsODNs into the cells and increase their inhibitory efficiency compared to free ODN at the same condition (20). The reason may be that ODN-containing liposomes are favorable for uptake by cells and the nanoparticulate carrier is able to increase AsODNs uptake by cells, especially through endocytosis (21). Free ODNs are both hydrophilic and big (MW > 5000) and cannot permeate the tight lipophilic cell membranes in the absence of liposomes sufficiently.

*Enhancement ability of IPM, cineole and limonene on liposomal gene delivery*

As it is seen in Figure 2. after 4 h pretreatment of A549 cells by IPM, the sequence specific toxicity of AsODN increased significantly (P<0.05) from 14% to 35% in a concentration dependent manner. At 5%, IPM showed nonspecific toxicity of about 40% which could be a combination effect of IPM and its solvent (DMSO). These findings are in agreement of Kasliwal et al. who showed that DMSO and IPM are potent chemical penetration enhancers (22). It is also in good agreement with Saffari *et al.* (20) who showed that lipid-fluidizing penetration enhancers are able to improve liposomal gene delivery. 

*Enhancement ability of IPM, cineole and limonene on liposomal gene delivery*

As it is seen in Figure 2. after 4 h pretreatment of A549 cells by IPM, the sequence specific toxicity of AsODN increased significantly (P<0.05) from 14% to 35% in a concentration dependent manner. At 5%, IPM showed nonspecific toxicity of about 40% which could be a combination effect of IPM and its solvent (DMSO). These findings are in agreement of Kasliwal et al. who showed that DMSO and IPM are potent chemical penetration enhancers (22). It is also in good agreement with Saffari *et al.* (20) who showed that lipid-fluidizing penetration enhancers are able to improve liposomal gene delivery. 

Figure 3. shows the effects of cineole and limonene on ODN cytotoxicity at 0.2 to 10 µg/mL. Results (Figure 3.) show that cineole significantly (P < 0.05) increased AsODN effect at higher concentration (2 and 10 µg/mL) while limonene didn’t affect the inhibitory effect of genetic material. The sequence specific cytotoxicity of liposomal AsODN was increased by cineole more than 150%. ScODN didn’t show significant toxicity (P > 0.05) even in presence of cineole or limonene as penetration enhancers. Studies on lamellar liquid crystalline structures, which resemble liposomes, by Moghimi *et al.* (18) demonstrated that limonene have weak enhancement effect toward permeation of hydrophilic drugs through lamellar lipid structures, while cineole showed up to 40 times enhancement effects on the same system (18). Cineole should be safe at these concentrations as its LD50 in oral administration to rat is reported to be 1280 mg/Kg (23).

Polarized light microscopy and differential scanning calorimetry studies on lipid bilayers by Moghimi *et al.* also showed that bilayer fluidization effect of cineole is more than limonene (18, 24). These findings are in agreement with the present results and show that selection of suitable enhancer can be useful in gene therapy and that penetration enhancers are helpful to increase transfection efficiency by liposomal gene delivery systems. 

## Conclusion

The present results show that it is possible to increase cellular uptake and biological effect of liposomal gene delivery systems by chemical penetration enhancers such as isopropyl myristate and cineole. Our results also show that the chemical enhancement effects are concentration dependent and also such effects are dependent on chemical structure of the enhancer. The results also reveal that lipophilic enhancers might displace genetic materials from the core of lipid bilayers in cationic liposomes. 

Further studies are in progress in our laboratories to investigate the enhancement effects of cineole at cellular levels and also to include such enhancers in our previously reported (25, 26) controlled release liposomal systems.
